# Autonomy without immunity: conventionality control and the limits of sporting sovereignty in the Inter-American system

**DOI:** 10.3389/fspor.2026.1949642

**Published:** 2026-10-23

**Authors:** Roberto Leonardo Cruz-Núñez, Hugo Armando Aguilar-Brito, Andherson J. Aguirre-Lanegra, Nuccia Seminario-Hurtado

**Affiliations:** 1 Centro de Estudios para la Construcción de Ciudadanía y la Seguridad, Universidad Autónoma de Chiapas, Tuxtla Gutiérrez, Mexico; 2 Universidad Tecnológica del Perú, Lima, Peru

**Keywords:** American Convention on Human Rights, conventionality control, due process, Latin America, lex sportiva, sport governance, sporting autonomy, sports arbitration

## Abstract

Professional athletes and clubs can compete only if they accept the jurisdiction of an arbitral tribunal seated outside their own country. International federations rely on that arrangement to claim immunity from judicial review; this article calls that claim sporting sovereignty. European courts have narrowed it by holding that compulsory sports arbitration must satisfy fair-trial guarantees, but protection there still depends on the courts of the state where the federation is seated and on a narrow reading of public policy, so the substance of a discrimination complaint may go unexamined. This article asks how far Articles 8 and 25 of the American Convention on Human Rights limit the rule-making authority of transnational sports organisations in states parties, through conventionality control, the duty of every domestic authority to test the rules it applies against the treaty. It proceeds by doctrinal legal analysis and by comparing the European and Inter-American standards of review, and it examines four jurisdictions: Peru, Colombia, Chile and Brazil. Inter-American judicial guarantees attach to the function a body exercises rather than to its formal character; they reach private power through the state's duty to ensure rights; and every domestic authority must apply them of its own motion, wherever the federation is seated. The comparison shows that Brazil and Chile channel contractual and employment disputes between player and club to the state courts, and that Colombia protects players through the constitutional action of tutela, so that closure is confined to disciplinary, eligibility and competition matters; Peru is the exception, having legislated the primacy of federation rules without such limits. In the Peruvian and Colombian cases examined, the delays that harmed a club and a player came from federation bodies overrunning their own deadlines, which undermines the argument that speed requires exemption from review. Sporting sovereignty is thus a delegated and conditioned authority. The article proposes a criterion of conditioned autonomy under which the power of federations is legitimate where it pursues a permissible aim, observes procedural guarantees, decides while a decision can still serve the person affected, and respects the substantive limit of human dignity and non-discrimination.

## 1 Introduction

Participation in organised professional sport requires, as a condition of access, acceptance of an arbitral jurisdiction situated outside the state in which the athlete lives and competes. International federations write into their statutes clauses that exclude recourse to the ordinary courts and refer disputes to the Court of Arbitration for Sport (hereinafter, CAS), seated in Switzerland, whose decisions become final under the law of that seat. Anyone who intends to compete accepts that referral without room for negotiation, since the alternative is exclusion from the activity for which they have trained. The legal question this arrangement raises is not whether specialised arbitration is desirable, since its usefulness for keeping the rules uniform is not in doubt. It is on what basis decisions taken within that circuit can be withdrawn from review by the state organs responsible for guaranteeing the rights of those who bear their effects.

Scholarship has described in detail how the transnational sporting order understands itself. Kolev (1) presents the *lex sportiva* as an anational body of rules comparable to the *lex mercatoria*; Casini (2) stresses its global reach; Duval (3) shows that it is ordered on quasi-constitutional lines around the Olympic Charter and the principle of the autonomy of sport; and Foster (4, 5) argues that a global sports law claiming immunity from state law conceals the continued self-regulation of the federations and has consolidated into an ideology. What that literature has not identified is the source from which the claimed immunity would derive.

The European Court of Human Rights (hereinafter, ECtHR) held Article 6 § 1 of the Convention applicable to sports arbitration and distinguished voluntary from compulsory arbitration; on that distinction, fair-trial guarantees cannot be waived where submission is imposed as a condition of professional practice (6), a criterion later extended to the arbitral bodies of national federations (7). The Grand Chamber went furthest. It described sport governing bodies as exercising powers close to those of a public authority and required a particularly rigorous examination by the reviewing court where arbitral jurisdiction has been imposed, while dismissing the substantive complaints of discrimination for want of a territorial link (8). Commentary on the earlier Chamber judgment had already stressed what the case meant for the relationship between sports arbitration and human rights (9), and commentary on the Grand Chamber ruling reads it as protection that stops short of the merits (10, 11). The Court of Justice of the European Union (hereinafter, CJEU) confirmed an equivalent limit shortly afterwards, admitting effective review of the award while confining it to conformity with the public policy of the Union (12). Both courts accepted the reasons ordinarily given for arbitral autonomy, the interest in a specialised body able to rule quickly and the value of handling disputes uniformly within a discipline (6, § 98; 12, § 94), and neither treated those reasons as a ground for dispensing with review. The combined result protects the procedure and leaves the substance to the jurisdiction of the state where the sporting organisation is seated.

Dulitzky (13) has shown that the CAS, when it turns to international human rights law, relies almost exclusively on the European Convention and the case law of Strasbourg, and that in the awards he examined none mentioned the Inter-American or the African systems. The scholarly literature shows the same bias: bibliometric analysis of the field reveals research concentrated in Europe and North America, with limited engagement from other regions and scant attention to the intersection between sports law and governance (14). The omission is striking because the Inter-American system holds resources that scholarship has not brought to bear on the problem, as the survey by Pérez González (15) of how international bodies have intervened in sporting matters makes clear. One judgment of the Inter-American Court of Human Rights (hereinafter, IACtHR) did arise from football, but its *ratio* concerns the delayed enforcement of a final judgment and belongs to a settled line on that subject (16); this article does not treat it as a precedent on the legal status of athletes. So far as our review extends, no work has connected the doctrine of conventionality control with the rule-making authority of transnational sports organisations.

Latin American athletes are subject to the same compulsory arbitration that European case law has brought under scrutiny, and they have not obtained the protection European litigants secured, although their states have assumed treaty obligations of broader scope than those flowing from the European Convention. Peru illustrates the tension most sharply, since a statute declares that the statutes, regulations and decisions of the Fédération Internationale de Football Association and of the Confederación Sudamericana de Fútbol prevail over any domestic rule. Other jurisdictions in the region have drawn the line elsewhere, confining the priority of federation rules to disciplinary and competition matters and sending contractual and employment disputes to the state courts, which is why the comparison matters. Access to sporting practice also involves an interest protected by international instruments, both through its recognition as a fundamental right without discrimination (17) and through its relation to public health, since physical inactivity ranks among the principal risk factors for premature mortality (18) and links the question to Sustainable Development Goal 3.

This article examines how far the guarantees of Articles 8 and 25 of the American Convention on Human Rights (hereinafter, American Convention), which protect the right to a fair trial and the right to judicial protection, limit the rule-making authority of transnational sports organisations in states parties, through the doctrine of conventionality control. It argues that sporting sovereignty is a delegated and conditioned authority, so that the finality of the arbitral award and the doctrine of non-interference yield to those guarantees; and that the Inter-American standard affords broader protection than the European one for three reasons, namely its material scope, which encompasses any proceeding determinative of rights; its rule of attribution, which places the obligation on the state irrespective of the organisation's seat; and its operability, which binds all domestic organs *ex officio*.

The analysis is doctrinal and conceptual. Its primary sources are treaties, the case law of regional human rights courts, sports arbitral awards, national legislation and constitutional judgments, complemented by specialised scholarship. The European and Inter-American models are compared at the level of rules, by contrasting the standards of review each imposes and the rules of attribution of responsibility each applies. Four jurisdictions in the region, Peru, Colombia, Chile and Brazil, are examined to illustrate the tensions identified; that examination grounds no empirical generalisation, since the study incorporates no original data and does not purport to measure how often the conflicts analysed occur. The standard applicable to sports arbitration is constructed by interpretation, from criteria the IACtHR has established for analogous situations, and the article indicates expressly where that projection goes beyond what the Court has so far decided.

## 2 Foundations and limits of sporting sovereignty

International sport is governed by rules of private origin that their own authors treat as a self-contained legal order of worldwide reach. Kolev (1) calls that *lex sportiva* anational and compares it with the *lex mercatoria*, the merchant law that grew up outside state legal systems; both claim to originate in a functional community that regulates itself without the national legislature. Casini (2) adds that sporting rules are global law in a fairly strict sense, since they apply everywhere, produce effects at the international and domestic levels alike and bear directly on individuals. On that account the Olympic Charter serves as a private instrument of constitutional character that states undertake to respect, and the World Anti-Doping Code supplies the framework for harmonising anti-doping policies, rules and regulations across sporting organisations.

Duval (3) has traced how that order understands itself in quasi-constitutional terms. The Olympic Charter works as a supreme norm exerting what he calls a centripetal force on the sub-constitutions of the international federations, while the Fundamental Principles of Olympism take on a constitutional role before the CAS, where they are invoked to challenge decisions of members of the Olympic Movement. Above the whole structure sits the autonomy of sport, which the International Olympic Committee relies on to claim independence from governmental interference and to reserve a sphere of functional sovereignty for the Olympic Movement. The term is a transposition: it applies to sport a category, sovereignty, coined for the ultimate power of the state.

That claim does not rest only on the will of those who make it; it answers a real problem in running competitions. Tournaments played simultaneously in dozens of jurisdictions need uniform rules applied in the same way, since divergent interpretation in each forum would make it pointless to compare results reached under different standards. A professional athlete's career is short, so a dispute settled after several years yields a nominal victory once the career is over. And the integrity of a championship requires challenges to be decided before results become definitive, because late correction disturbs standings, promotions and relegations already acted upon. Sports law has a name for this cluster of concerns, the *pro competitione* principle, and specialised arbitration has written it into its own rules. The CAS Code allows an expedited procedure in ordinary arbitration with the consent of the parties (19, art. R44.4) and in appeals arbitration with their agreement (art. R52), and it provides for abbreviated time limits on provisional measures and, in cases of utmost urgency, for an order on the mere presentation of the application, the other party being heard afterwards (art. R37).

Put at its strongest, the objection to state review runs as follows. If speed and uniformity are preconditions of international competition, subjecting federation decisions to state courts would introduce delay and divergent interpretations that defeat what the sporting order exists to achieve, at the expense of the very athlete the system claims to protect. The answer comes from the courts that have gone furthest in scrutinising sports arbitration. The ECtHR said as much in *Mutu and Pechstein*: there is a real interest, it held, in referring disputes arising in professional sport, particularly those with an international dimension, to a specialised body able to rule quickly and inexpensively, and a single specialised international arbitral tribunal makes for procedural uniformity and reinforces legal certainty (6, § 98). The Grand Chamber repeated the point, holding that arbitration imposed by a private entity rather than by law does not in itself give rise to a breach of Article 6 § 1 (8, § 199). The CJEU made the same concession by its own route: imposed arbitration, it accepted, may in principle be warranted by the legal autonomy of international sports associations and by their responsibilities, where it pursues legitimate objectives such as uniform handling of disputes within a discipline or consistent interpretation and application of its rules (12, § 94). Both courts accepted the premise and both still required effective judicial review of the award.

What needs justifying is the step from autonomy to immunity from state law, and those who defend immunity rarely make that step explicit. Foster (4) supplies the distinction that isolates the problem: alongside an international sports law that national courts can apply, there is a global sports law claiming immunity from national law. That global law, on his reading, conceals the continuing self-regulation of the international federations and works against the rule of law in sport. His later reassessment goes further: autonomy has settled into an ideology under which the sporting order counts as a self-sufficient transnational legal system, and that belief has allowed sporting bodies to claim effective immunity from review by national courts and to keep a form of self-government insulated from accountability (5). The criticism now has judicial support. Immediately after acknowledging those legitimate objectives, the CJEU recalled that the legal autonomy of international sports associations cannot justify their exercising their powers so as to restrict the possibility for individuals to rely on rights and freedoms conferred by EU law which form part of EU public policy, and that this requirement means that respect for those rights must be open to effective judicial review, *a fortiori* where arbitration is imposed on those concerned (12, § 95).

The immunity claim also sits badly with the internal composition of the *lex sportiva*, which is far from homogeneous. Foster (20), having examined the awards of the CAS, finds its case law organised around five heterogeneous criteria (a *lex ludica* attentive to the rules of the game, good governance, procedural fairness, harmonisation of standards among federations and equal treatment) and concludes that the label itself is imprecise and covers disparate notions. One consequence follows: procedural fairness already figures among the principles the tribunal applies of its own motion, so the sporting order itself acknowledges that correctness of procedure matters. External review of due process cannot be presented as an intrusion foreign to its logic when the same requirement already governs within its own repertoire.

The same pairing appears in the most developed national codification of the field. Article 2 of the Brazilian Code of Sports Justice (*Código Brasileiro de Justiça Desportiva*) lists the principles governing its interpretation and application and places in a single series the prevalence, continuity and stability of competitions, expressly labelled *pro competitione*, alongside due process of law, the requirement that sporting offences be defined in advance and sportsmanship (21). The Brazilian rule-maker did not treat the speed of competition as a value that displaces procedural guarantees; both appear in the same provision and at the same rank. Whatever exclusivity is claimed for *pro competitione*, it finds no support in the positive sports law that enshrines the principle.

Duval (22) describes the method of the CAS as assemblage: the tribunal combines Swiss law, EU law and the European Convention on Human Rights to produce awards that carry authority, and what characterises its practice is the hybridity of its materials rather than the purity and autonomy proclaimed in official discourse. A legal order that by construction draws on state law, regional law and human rights law cannot, without contradiction, claim hermetic closure against those same sources.

The discourse of sporting sovereignty runs together three things that are better kept apart. The autonomy of the sporting order is a fact: its rules govern a community of worldwide reach, they are structured on quasi-constitutional lines and its disputes go to a specialised arbitral body. The functional reasons supporting that autonomy deserve equal weight, because uniform rules and rapid decisions protect legitimate interests, the athlete's among them. Immunity from judicial and constitutional review is a different matter, and it follows from neither premise. That a legal order regulates itself effectively, and has good reasons to do so quickly, does not release it from answering to the standards that protect those subject to its decisions. Examined at its foundations, the sovereignty that sport claims for itself is a delegated and porous authority, dependent on the sources that feed it.

## 3 Scope and limits of European review of sports arbitration

The immunity that the sporting order claims for itself began to give way before the European courts through a distinction with lasting consequences. In *Mutu and Pechstein* the ECtHR held Article 6 § 1 applicable to CAS arbitration and separated voluntary from compulsory arbitration, the latter being arbitration required by law, which must afford the safeguards secured by that provision (6, § 95). Applying that distinction to Claudia Pechstein, the Court asked what refusing the arbitration clause would have meant for her professional life and concluded that she could not be said to have accepted it freely and unequivocally (§ 114); her acceptance of CAS jurisdiction counted as compulsory arbitration, even though the imposition came from the regulations of the International Skating Union rather than from law (§ 115). Not every complaint succeeded. The Court rejected the challenge to the independence and impartiality of the CAS (§ 159) and found a violation only in the refusal of a public hearing, which the Swiss Federal Supreme Court had denied on the premise, rejected in Strasbourg, that Article 6 did not apply to voluntary arbitration (§ 183).

Two years later the same requirement reached the arbitral bodies of national federations. In *Ali Rıza and Others v. Turkey* the Court identified strong organisational and structural ties between the Board of Directors of the Turkish Football Federation and its Arbitration Committee, ties that gave the Board considerable influence over how the Committee functioned (7, § 216). It also found that players did not enjoy the same level of representation as clubs in the Federation's governing organs, which could tip the balance in favour of clubs in contractual disputes heard by the Committee (§ 219). Absent safeguards against outside pressure, the applicants had legitimate reason to doubt the Committee's independence, and Article 6 § 1 had been violated (§ 223). The Court did not stop at individual redress: acting under Article 46, it called for general measures to reform the system for settling football disputes under the auspices of the Federation. Scrutiny of fair-trial guarantees had reached the domestic federation level, and with it the state's responsibility for how those internal tribunals are composed.

*Semenya v. Switzerland*, decided by the Grand Chamber in 2025, takes the line as far as it has gone and shows where it stops. The Court first accepted the model: a non-state mechanism of dispute resolution at first or second instance, with an appeal, limited though it may be, to a state court at last instance, could be an appropriate solution in this field (8, § 199). A reservation follows at once. Sports arbitration takes place against the structural imbalance that often marks the relationship between athletes and the bodies governing their sport, and the Swiss Federal Supreme Court has itself acknowledged that competitive sport is organised in a strongly hierarchical way, so that relations between athletes and governing bodies run along a vertical axis rather than the horizontal one of contracting parties (§ 200).

The Grand Chamber then described the power at issue in this study more precisely than any court had before. Sport governing bodies exercise powers in international competition akin to regulation-making powers, determining who may compete and on what conditions, and the imposition of the CAS's mandatory and exclusive jurisdiction forms part of the exercise of those powers (§ 201). Private entities not governed by public law, the Court concluded, *de facto* regulate individual activity and can restrict the exercise of rights, exercising powers close to those of a public body (§ 202). It traced the structural dominance behind that position: the CAS was created under the auspices of the International Olympic Committee, and the International Council of Arbitration for Sport, whose members are designated directly or indirectly by the Olympic bodies, adopts the Code of Sports-related Arbitration, appoints the listed arbitrators and finances the CAS (§ 203). Governing bodies are placed to dictate the terms of their relationship with athletes (§ 204).

The Court went on to hold that an athlete on whom the CAS's exclusive jurisdiction has been imposed must be able to rely on the guarantees of Article 6 § 1 even more than where arbitration is required by law (§ 205). Imposition by a private body thus calls for closer scrutiny, whereas the specificity of sport is often invoked to justify less. The requirement is stronger still where the civil rights in dispute correspond in domestic law to fundamental rights (§ 206). Where the CAS's mandatory and exclusive jurisdiction has been imposed, where the dispute concerns civil rights and where those rights correspond to fundamental rights, a fair hearing requires a particularly rigorous examination of the athlete's case (§ 209). The Swiss Federal Supreme Court had not conducted it, applying to CAS awards the same restrictive approach to public policy that it follows in commercial arbitration, and Article 6 § 1 was violated on that account, by fifteen votes to two.

The second holding marks the boundary of European protection. By thirteen votes to four, the complaints under Articles 8, 13 and 14 were dismissed as incompatible *ratione personae* and *ratione loci* for want of a territorial link with Switzerland: the contested regulations had been adopted by World Athletics from its seat in Monaco, and the applicant holds South African nationality. That disposal reversed the Chamber, which in 2023 had found a violation of Article 14 taken with Article 8 by four votes to three, and a violation of Article 13 by the same majority (23). A rigorous procedure was secured; the substance of the discrimination alleged went unexamined, because protection turned on the territorial jurisdiction of the state where the award was challenged. Commentary on the Grand Chamber judgment reads it as a missed opportunity for the protection of rights in sport (10) and contrasts the procedural rigour required with the substance left unexamined (11).

EU law confirmed the same boundary from another direction. In *Seraing* the CJEU found that the CAS award had been made under an arbitration mechanism which, in practice, must be regarded as unilaterally imposed on those concerned (12, § 96), and it grounded that finding in the FIFA Statutes then applicable: Articles 47(3) and 50(1) required appeals against final FIFA decisions to be lodged with the CAS, and Article 50(4) denied those appeals suspensive effect unless the CAS granted it (§ 97). Where no direct remedy against the award exists, the individuals concerned must be able to obtain indirect review, on their own application or by the court's own motion, before any court of a Member State that may examine the award in any manner whatsoever, and a Member State that provides no such avenue must create one (§ 100). That review extends to the interpretation of the principles and provisions of EU public policy, to the legal consequences of that interpretation and to the legal classification of the facts established by the arbitral body (§ 101). Nor may the reviewing court stop at declaring an inconsistency; it must be able to draw all the appropriate legal conclusions, since review that leaves the inconsistency standing would not be effective (§ 103). The operative part follows: *res judicata* may not be conferred within a Member State on a CAS award whose consistency with EU public policy has not first undergone effective review by a court empowered to make a preliminary reference. Boddin (24) reads the ruling as leaving mandatory arbitration itself largely untouched while tightening the standard of review. The narrowness of that avenue is confirmed by the statistics available for the period up to 2015: challenges before the Swiss Federal Supreme Court succeeded in roughly ten per cent of sports cases against some seven per cent elsewhere, and only two requests for revision were granted out of twenty-three published rulings (25).

European review has limited the immunity that the sporting order claimed and has brought both the CAS and national federation tribunals within fair-trial guarantees; it has also described, with unusual precision, private bodies wielding powers close to those of a public authority. What legitimates the model in the Court's own account is the availability of effective judicial review at last instance. That condition is satisfied only in form when the review is confined to the public policy of a single state, read narrowly, and when the athlete's substantive complaints fall outside the reviewing state's jurisdiction because the governing body is seated elsewhere. The guarantee then depends on where the power that imposed the arbitration happens to be domiciled, rather than on the weight of the rights of the person subject to it.

## 4 The Inter-American standard of protection against sports arbitration

When the CAS turns to international human rights law, its repertoire narrows to the European Convention and the case law of Strasbourg; in the awards Dulitzky (13) examined, none mentioned the Inter-American or the African systems, although wholly African disputes had been decided by European standards, which suggests a choice rather than a necessity. Ibero-American scholarship has surveyed the intervention of international bodies in sporting matters and found the United Nations treaty-body route underused, while the regional system offers more examples, among them Report No. 48/96 of the Inter-American Commission on Human Rights, which declared inadmissible, for failure to exhaust domestic remedies, a petition alleging sex discrimination in the prizes awarded at municipal athletics competitions in Costa Rica (15).

One judgment of the IACtHR arose from football, and its use here should be stated precisely. In *Meza v. Ecuador* a professional footballer obtained a final judgment against his club and waited more than a decade for it to be enforced. The *ratio* concerns that delay, not the jurisdictional regime to which players are subject: the Court grounded the state's duty in Article 25(2)(c) of the American Convention, which guarantees compliance by the competent authorities with any decision granting a remedy (16, § 59). The case belongs to a line on the enforcement of final judgments that runs through *Mejía Idrovo v. Ecuador* (26), *Furlan and family v. Argentina* (27), *Muelle Flores v. Peru* (28) and *Teachers of Chañaral v. Chile* (29), and it is no authority on the legal status of athletes. What it does supply, alongside that line, is examined below; nothing in the argument depends on treating it as a precedent about sport.

The first element of the model lies in how broadly the American Convention conceives the guarantees of Article 8, which the Court calls judicial guarantees. Deciding the case of the Panamanian public employees dismissed under a retroactive statute, the Court held that obtaining all the guarantees that make fair decisions possible is a human right, and that the administration is not exempt from that duty; the minimum guarantees must be observed in administrative proceedings and in any other procedure whose decisions may affect rights (30, § 127). Two days earlier it had held that any state organ exercising functions of a materially adjudicative character must take decisions consistent with due process under Article 8 (31, § 71). The guarantee attaches to the function exercised rather than to the name of the body exercising it, which covers both an arbitral panel deciding a sporting appeal and a federation's disciplinary organ. *Meza* confirms the breadth: Article 8(1) governs the determination of rights and obligations of a criminal, civil, labour, fiscal or any other nature, and the Court applied it to a dispute between a footballer and his club (16, § 61).

The second element is the mechanism that makes those guarantees effective inside the state. In *Almonacid Arellano* the Court established that judges are bound by the American Convention on ratification and must prevent its effects being defeated by laws contrary to its object and purpose, exercising what it termed a kind of conventionality control between domestic norms and the treaty, taking into account the treaty and also the interpretation given to it by the Inter-American Court (32, § 124). *Cabrera García and Montiel Flores* extended the duty to judges and organs linked to the administration of justice at every level, acting *ex officio* within their competences, all the organs of a state party being bound by the treaty (33, § 225). *Gelman* went further: conventionality control, the Court held, is a function and task of any public authority and not only of the judiciary (34, § 239). Ferrer Mac-Gregor (35) reads the doctrine as a duty incumbent on all national judges, exercised with different degrees of intensity according to their competences and procedural rules. For the problem examined here the consequence is direct: the duty falls on any domestic authority applying a norm, whatever the public or private origin of the provision, once the state legal order has incorporated or recognised it.

It might still be said that federations and arbitral bodies are private entities and so foreign to a treaty binding on states. The Court met that objection in its Advisory Opinion on undocumented migrants. In an employment relationship governed by private law, it held, an obligation to respect human rights exists between private parties, because the state's positive duty to ensure protected rights produces effects towards third parties; and it linked that obligation to the theory of *Drittwirkung*, under which fundamental rights bind public authorities and private parties alike in their relations with one another (36, § 140). States determine the legal order regulating such relations, and must ensure that rights are respected within them, failing which they incur responsibility (§ 147). Scholarship has systematised that horizontal effect and shown that it works through the state's duty to ensure rights, whose breach engages domestic and international responsibility (37), and comparative constitutional analysis in the region treats the same doctrine as positive law where a constitution binds every person, institution and group (38).

That horizontal reach is not confined to advisory opinions. It has been worked out in contentious cases on the enforcement of final judgments, the line to which *Meza* belongs. In *Teachers of Chañaral* the Court held unanimously that Chile had violated Articles 8(1), 21, 25(1) and 25(2)(c) in respect of 846 teachers holding final judgments against municipalities. Article 25(2)(c), it recalled, enshrines the right to compliance by the competent authorities with any decision granting a remedy (29, § 142), and state responsibility does not end when a court gives judgment, since enforcement must be “complete, perfect, integral and without delay” (§ 143). *Muelle Flores* had already applied the principle where the debtor was a privatised company: even assuming a private entity was liable for the pension, the state was obliged to ensure that it was paid (28). Building on both, the Court stated in *Meza* that the guarantee of effective enforcement mechanisms applies to final decisions rendered against state entities and against private parties alike (16, § 60), and its own footnote traces that statement to *Muelle Flores* and *Chañaral*. The proposition rests on a settled line, then, rather than on an isolated dictum in a case that happened to involve a football club.

A further regional development supports the analogy. Building on *Baena Ricardo*, Latin American courts have extended the guarantees of Articles 8 and 25 to administrative proceedings, a development Hachem (39) calls the conventionalisation of administrative law, which requires such legislation to be reread against treaty standards. If the administration's sanctioning powers fell under fair-trial guarantees although they are not exercised before a court, the disciplinary power of a sporting federation cannot claim gentler treatment: it is functionally equivalent, imposing sanctions that affect professional activity and assets. What matters is the weight of the rights affected by the sanction, not the nature of the body imposing it.

In states parties to the American Convention, then, the finality of a sports arbitral award and the non-interference doctrine that international federations invoke against public authorities yield to the minimum guarantees of Articles 8 and 25, whose observance every domestic organ must secure through conventionality control. Its protective reach exceeds that of the European standard in three respects. First, Article 8(1) applies to the determination of rights and obligations of a civil, labour, fiscal or any other nature, whereas Article 6 of the European Convention covers civil rights and obligations and criminal charges, and the prohibition of discrimination reaches private power through Articles 1(1) and 24 of the American Convention rather than through the procedural provision alone. Second, the obligation falls on the state party in respect of those within its jurisdiction, wherever the federation is seated, which removes the *ratione loci* barrier that stopped the Grand Chamber from reaching the merits in *Semenya*. Third, conventionality control binds every state organ *ex officio*, and does not wait on a supreme court willing to read its public policy broadly.

Conventionality control has been criticised for its expansive reach and for the authority it gives the Inter-American Court over domestic legal orders, with the risk of eroding national democratic deliberation (40, 41), and the allocation of competence between the regional and the national level remains contested (42). A second objection is technical: horizontal effect makes the state responsible for failing to ensure rights between private parties, and generates no direct international claim against the federation. Neither observation disables the model. Those replying to Contesse's first article maintain that it mischaracterises one crucial technical component of the doctrine, with different conclusions following once that component is read correctly (43), and Ferrer Mac-Gregor (35), one of the doctrine's principal exponents, denies that the Inter-American Court defines or imposes the systems of constitutional review that each country adopts. Even on its most cautious reading, Articles 8 and 25 bind every state organ. The second objection clarifies the mechanism and makes it more workable: what the model asks of domestic courts is review of sporting decisions, or refusal to enforce them, where they deprive a person of the guarantees of the Convention, without requiring the Court to adjudicate against an international federation. *Meza* confirms that route. Responsibility there was declared against the state for the conduct of its judicial organs in a dispute with a sporting club, and the Court dismissed the fourth-instance objection by recalling that examining the compatibility of judicial acts with the Convention is not an additional tier of review (16, §§ 17 and 18). The finding of a violation of Articles 8(1) and 25 was reached by five votes to two, over a joint partly dissenting opinion holding the delay not attributable to the state.

Examined under the American Convention, sporting sovereignty becomes a conditioned authority, because the guarantee no longer depends on where the organisation issuing the norm is domiciled but on the obligations the state assumed towards those within its jurisdiction. The practical effect is considerable: a Latin American athlete may ask their own courts to examine decisions that affect them, without being sent to the courts of the state where the federation chose its seat or to the reading of public policy prevailing there. Such examination happens only where an authority is willing to undertake it and legislation does not stand in the way. Whether the model works is decided within the particular legal system where the transnational sporting norm and the guarantee under the Convention come into conflict.

## 5 Testing the standard in the jurisdictions of the region

Peruvian law entrusts the governance of sport to a public body. Law No. 28036 designates the Peruvian Institute of Sport (hereinafter, IPD) as the governing entity of the National Sports System, a decentralised public body with ministerial rank attached to the Presidency of the Council of Ministers and endowed with technical, functional and administrative autonomy (44, Article 7). In 2018 that arrangement was inverted for the country's most prominent sport. Law No. 30727 declares the Peruvian Football Federation (hereinafter, FPF) the governing body of national football, subject to its own by-laws and to that law, and also to the statutes, regulations and decisions of the Fédération Internationale de Football Association and the Confederación Sudamericana de Fútbol, which prevail over any domestic rule (45, Article 1). The following provision grants the FPF full autonomy in sporting, administrative, economic, financial and organisational matters and in the settlement of disputes within its competence (Article 2), while Article 5 reduces its relationship with the IPD to informing it of the calendar of activities. The legislature did more than recognise self-government by the federation: it gave a private body of foreign rules primacy over domestic law and dismantled the oversight it had itself created. The IPD later declared that the FPF no longer fell within Law No. 28036 or under its own supervision.

In September 2018 a bill was introduced to amend Law No. 30727, and on 1 October 2018 the Committee on Education, Youth and Sport of the Congress heard the FPF and the Confederación Sudamericana de Fútbol on it. According to the Congress's own news service, the FPF's secretary general warned that if the domestic legal order did not allow the federation's statutes to conform to FIFA's requirements, a sanctions procedure could follow that might end in suspension, and that FIFA might reconsider awarding the under-17 World Cup to Peru; the Deputy Secretary General of the Confederación stated at the same hearing that her institution exercised no interference in the FPF's decisions (46). The structural coercion that European case law identified when denying the voluntary character of arbitral submission reappears here one level up, in the legislature, which considered amending the statute under a threat to exclude its national team and its clubs from international competition.

Other jurisdictions in the region have drawn the line differently, and the Brazilian solution is the most precise. Article 217 of the 1988 Constitution recognises the autonomy of sporting bodies in their organisation and functioning (47, item I) and then confines the consequences of that autonomy. The courts may hear actions relating to sporting discipline and competitions only once the instances of sporting justice (*justiça desportiva*, the disciplinary tribunals of the sporting bodies) have been exhausted, and sporting justice must decide within sixty days of the proceedings being opened (Article 217, §§ 1 and 2). Two limits are built into the text. Prior exhaustion covers disciplinary and competition matters alone, so anything else goes straight to the courts, and the exemption is temporary by design, since a body that does not decide within sixty days loses it. The Pelé Act follows the same logic: sporting justice is limited to the processing and adjudication of disciplinary infringements and competitions (48, Article 50), its tribunals must always guarantee the right to a full defence (*ampla defesa*) and the adversarial principle (Article 52), and the professional athlete's activity rests on a special sports employment contract (Article 28). The General Sports Act of 2023 now regulates that contract for every sport (49, Article 85), while the Pelé Act remains partly in force because its repeal was vetoed. The Superior Labour Court drew the conclusion in 2009: Article 29 of Law No. 6,354/76, which required sporting instances to be exhausted before a labour claim could be brought, was held incompatible with the 1988 Constitution and therefore not carried over into the new constitutional order, because only disciplinary and competition matters fall under Article 217, § 1 (50).

Chile reaches a comparable result by statute. Law No. 20,178 inserted into the Labour Code a chapter governing the employment relationship, under subordination, of those engaged in professional football and of workers in connected activities (51, Articles 152 bis A and 152 bis B). Players litigate against clubs as employees, and they do so in practice. In 2018 Mauricio Pinilla brought a claim against Azul Azul S.A. under the labour procedure for the protection of fundamental rights (*tutela laboral*) introduced by Law No. 20,087 (52). The Labour Court of San Miguel upheld it, finding a breach of Article 19, paragraphs 4 and 16, of the Constitution and awarding the additional compensation provided for in Article 489 of the Labour Code; both parties then sought annulment before the Court of Appeal of San Miguel, where the player invoked Article 11(1) of the American Convention among other provisions, and the Court of Appeal set the judgment aside (53). The claim did not succeed in the end, yet the case is instructive on two counts: an athlete in the region has already argued the American Convention against a club, and the avenue that carried him there was the ordinary labour jurisdiction. Chilean scholarship describes the disciplinary and competition side, documenting how FIFA and the national federation have worked to keep sporting disputes away from the ordinary courts (54).

A distinction emerges that the argument needs. Contractual and employment disputes between player and club are heard by state courts in Brazil and Chile, whether under a special labour chapter or the ordinary labour jurisdiction, and in Colombia the constitutional action of tutela reaches them. Closure operates in disciplinary, eligibility and competition matters. Peru is the only one of the jurisdictions examined here that legislated the primacy of federation rules without confining it to those matters, and the consequences surfaced in litigation. Peruvian scholarship had supplied the rationale for the statute: as Varsi Rospigliosi (55) records, Seoane Linares had argued that the FPF, as a private-law non-profit association, runs its affairs like any other association, without interference, its assets being private and subject to audit only as any taxpayer's would be unless it receives public funds (Seoane Linares, as cited in 55). On that premise the statute could present itself as recognising an associative reality rather than ceding rule-making power.

The decisive case was an *amparo*, the Peruvian constitutional action for the protection of rights. Club Escuela Municipal Deportivo Binacional brought it against the FPF over final sanctions imposed on another club, Sport Boys Association: a fine, a warning and a cumulative deduction of points, all for breaches of financial criteria during 2022. The Federation failed to apply those sanctions in full to the table of the 2023 Liga 1 championship, and the omission left the claimant relegated. The delay is documented: the licensing report was notified to the penalised club on 28 October 2022 and forwarded to the next instance only on 10 March 2023. The Constitutional Court of Peru did not avoid the structural problem behind the dispute. It observed in international federations a marked tendency to remove their disputes from ordinary judicial review, including through clauses providing for severe sanctions such as expulsion or exclusion from all competition, and it reproduced Article 51 of the FIFA Statutes, under which recourse to the ordinary courts is prohibited unless FIFA's own rules provide otherwise (56, para. 21; the judgment does not identify the edition of the Statutes it quotes). Paragraph numbers here refer to the numbered legal grounds (*fundamentos*) of the judgment.

As a matter of principle, the Court was unequivocal. No constitutional justification exists for relieving the FPF of constitutional review where it fails to respect fundamental rights in the exercise of due process and effective judicial protection (para. 26). The Federation, like any other legal person governed by private law, must act within the lines set by the Constitution, so that its acts hold good only in so far as they do not contradict the values, principles and rights the Constitution recognises (para. 27). The decisive reasoning borrows the very category on which the Inter-American model rests: the horizontal effect of fundamental rights, which follows from their objective dimension, makes their effectiveness enforceable against private parties in their interpersonal relations and is an implicit limit, a requirement that flows from the state's primary duty under Article 44 of the Constitution to guarantee the full effectiveness of human rights (para. 28). From this the Court refused to accept spheres exempt from review under cover of organisational autonomy or private self-determination, since allowing rights to be relativised according to the sphere in which they are exercised would undermine the rule of law itself (para. 29).

What carried the claim was a drafting defect in the regulations rather than any judgement about sport. Article 104.3 of the FPF Licensing Regulations allows enforcement of a disciplinary decision to be suspended where the penalised club alleges harm that is difficult or impossible to repair, and provides no equivalent remedy for clubs adversely affected when that non-enforcement becomes permanent (para. 36). The judgment upheld the claim in part for breach of the right to due process, ordered the Federation to update the table of the 2023 first-division championship in line with the sanctions imposed, held that the claimant retained first-division status and was to be included in the Liga 1 tournament for 2027, and awarded costs against the Federation. The ruling was not unanimous. Justice Gutiérrez Ticse concurred in the result while arguing that sport is a fundamental right on the basis of Article 38 of the 1979 Constitution, the first to regulate it expressly; Justices Domínguez Haro and Morales Saravia dissented, holding the claim *improcedente*, that is, barred on procedural grounds, because the matter had become moot; and Justice Monteagudo Valdez set out separate considerations to the same dismissive effect. Subjecting federations to constitutional review is plainly not yet settled doctrine, although the disagreement ran on procedural rather than substantive lines.

Colombian case law has gone further in the same direction, and it started early. Reviewing the provisions of Law 181 of 1995 on sporting rights, the Constitutional Court struck out the word “exclusive” from the rule vesting those rights in clubs and upheld the remainder on the understanding that players may hold their own sporting rights, since preventing them from doing so tends to turn the player into a mere business asset and offends dignity, autonomy and the freedom to choose an occupation (57). In Judgment T-464 of 2022 the Court protected professional footballers whom the Colombian Football Federation and DIMAYOR (División Mayor del Fútbol Colombiano) had prevented from registering with a club, holding that the minimum self-determination of those private entities is reasonably limited by the free exercise of the players’ fundamental rights, which bars treating players as a resource for the undertaking's ends (58). Judgment SU-386 of 2023, a unification judgment by which the full Court settles the case law of its chambers, completed the picture: autonomy and organisational freedom must be exercised with respect for human dignity and the constitutional duties that Article 95 assigns to everyone, and the Ministry of Sport is competent to inspect and supervise the regulations of sporting organisations without thereby obstructing fundamental rights (59). An earlier decision speaks directly to the objection considered in this article. In Judgment T-740 of 2010 the Court held the tutela admissible to resolve contractual disputes between footballers and clubs, and the criterion it relied on was the short sporting life of the player, particularly where the dispute restricts transfers and the claimant is a minor (60). Ordinary legal actions against a club's decisions, it added, are not an adequate judicial remedy for the immediate protection of fundamental rights, and reducing a footballer to an asset of the club disregards those rights. The Court then found a breach of due process by the Player Status Committee of DIMAYOR, which had exceeded without justification the time limits its own Player Statute laid down.

That last finding reverses the argument usually built on the specificity of sport. Speed matters, and for the reason given: careers are short and a remedy that arrives late is no remedy. In the two cases examined here, it was the federation system that failed the test. The Colombian federation body overran its own regulatory deadlines; the Peruvian Federation left final sanctions unenforced until the competition had moved on. The *pro competitione* principle, applied with any rigour, tells against the bodies that invoke it. Brazil shows how the two concerns fit together: sporting justice keeps its priority over the courts in disciplinary and competition matters, and keeps it only for sixty days.

Both the Peruvian and the Colombian courts reached the result the Inter-American standard prescribes, and neither took the route that sustains it. The judgments examined make no use of conventionality control. A search of the Peruvian judgment returns no mention of the American Convention, of the Inter-American Court or of conventionality control, and its two references to human rights point to Article 44 of the domestic Constitution. Law No. 30727 went unexamined, and its Article 1 still gives the rules of international federations primacy over any domestic provision. Three consequences follow. The decision remains open to being presented before international bodies as state interference in the affairs of a federation, which is the very reproach that triggers the sanctions set out in the international statutes, and the court had no international obligation of its own state to set against that reproach. The statutory clause that produced the collision survives, so the conflict was resolved for one claimant and left standing at its source. And the criterion binds as domestic precedent alone, without activating the duty that Inter-American case law imposes on every state organ.

The Peruvian experience confirms that the model works and shows what it still lacks. A constitutional court can confront a clause excluding ordinary jurisdiction and hold a federation to due process on the strength of its own Constitution. That footing leaves the decision exposed, because the conflict is then framed as one between the domestic law of a single state and the rules of a transnational private organisation, and against that framing the threat of competitive exclusion keeps its force. Invoking the Convention standard changes those terms: it moves the basis of the decision from the sovereign will of one state to an international obligation shared by all the states of the region, disregard of which engages responsibility and cannot be described as interference. What was missing in both jurisdictions was available in the treaty that binds them.

## 6 Configuring conditioned sporting autonomy

The preceding sections warrant replacing the category of sporting sovereignty with one of sharper outline. The authority of sporting organisations is legitimate where three cumulative requirements are met: (i) the pursuit of a permissible aim, which covers the organisation of competition and the preservation of equal conditions among those taking part; (ii) observance of procedural guarantees in every decision bearing on rights, with the scope recognised by Articles 8 and 25 of the American Convention, and a ruling delivered while it is still of use to the person affected; and (iii) respect for a substantive limit made up of human dignity and the prohibition of discrimination. The second and third requirements follow from the standard examined earlier; the first captures the rationale of the power of federations. Autonomy so defined ceases to be an exception to the legal order and comes to be measured by its function.

Speed belongs inside the second requirement rather than outside the scheme, although it is often set against judicial review. A decision that arrives after the competition has closed, or after the career it concerned has ended, fails the guarantee it purports to serve. Nothing in this reading denies the value of quick, uniform resolution. It denies only that speed is obtained by removing review, and the record examined in the previous section suggests otherwise, since the delays that mattered there came from federation bodies overrunning their own deadlines.

The third requirement raises a difficulty that has to be faced directly. Review of sporting decisions, it may be said, must stay with procedure and leave the content of eligibility rules alone, because those rules express technical findings foreign to value judgement, and reviewing them would hand the judge a subject matter outside their competence. The objection has weight, and it goes a long way towards explaining the outcome before the European courts, where protection stopped at the rigour of the examination and never reached the substance of the discrimination alleged. Its force depends on a premise that can be tested: that eligibility rules really do rest on neutral technical criteria.

The anti-doping regime allows that premise to be tested, since the spirit of sport is one of the criteria on which a substance may be added to the prohibited list, and its indeterminacy has prompted the objection that a notion so vague should be discarded. McNamee (61) answers that objection with force, pointing out that it assumes a theory of meaning on which using a concept correctly would require necessary and jointly sufficient conditions. Wittgenstein's remarks on family resemblances showed that many terms lack a common core and are applied through partial similarities, without their openness impeding competent use. Hart's notion of defeasibility points the same way for legal concepts, law working with categories of clear outline and with others less clear. Vagueness alone, then, does not disqualify a normative criterion. Conceding that much leads to the conclusion the objection was meant to avoid. The systematic review by Obasa and Borry (62) finds that the multivalence of the spirit of sport is its most striking result, makes contestability the dominant theme of the literature surveyed and adds that empirical research is needed to capture the perspectives of other stakeholders, the athletes above all. Loland (63) identifies the point that matters here: the usual justifications for prohibition, built on fairness and on harm to health, are inadequate on their own and make sense only within a normative understanding of athletic performance and of the value of sport. A criterion that needs a substantive conception of sport in order to work yields decisions that embody evaluative choices, and they can be examined as such.

Philosophical criticism meets legal criticism at this point. Geeraets (64) examines the two justificatory arguments by which the World Anti-Doping Agency sustains the severity of its regime: that protecting the spirit of sport warrants tough measures, and that athletes have voluntarily consented to the Code. Neither withstands scrutiny as presented. Both, he argues, function ideologically, their aim being less to be correct than to ward off criticism, a reading He (65) develops in the same direction. The consent argument fails for the structural reason that deprived arbitral submission of voluntariness in European case law, and that weighed on the Peruvian legislature when it considered the statute under threat of international exclusion: someone who must accept a condition in order to practise their profession does not consent in any meaningful sense. That convergence matters, because consent is the standard defence of the sporting order against outside review, and it gives way both to conceptual analysis and to judicial scrutiny.

Litigation over prostheses makes the point most clearly, and does so where the appearance of scientific controversy is strongest. In the matter examined by Cornelius (66), the Council of the International Association of Athletics Federations, now World Athletics, ruled in January 2008 that the prostheses used by a double-amputee athlete were a technical device giving him an advantage over able-bodied competitors, contrary to Competition Rule 144.2(e), and the CAS set that decision aside. The later award concerning Blake Leeper makes the lesson more precise. The panel invalidated the rule that placed on the athlete with a disability the burden of showing that his mechanical aid conferred no overall competitive advantage and shifted that burden to the international federation; Viret (67), commenting on the award, observes that evidentiary questions prove decisive for the real-life impact of a policy involving complex scientific matters, to the point of forcing a revision of the regulatory approach. No biomechanical datum dictates who bears the burden of proof. That allocation is an institutional choice with distributive consequences, and whoever makes it decides who carries the risk of scientific uncertainty. Such choices are the ordinary exercise of the quasi-regulatory power the Grand Chamber described in *Semenya*, where private bodies not governed by public law were found to regulate individual activity *de facto* and to be able to restrict the exercise of rights (8, § 202).

The remaining question is which legal system supplies the substantive standard these disputes require, and the Swiss material shows why the answer is not obvious. On the Grand Chamber's own account, the case law of the Swiss Federal Supreme Court treats an award as incompatible with substantive public policy where it seriously breaches fundamental principles of substantive law, among them respect for human dignity and the personality rights set out in Articles 27 *et seq.* of the Swiss Civil Code. That court said as much in *Semenya* itself, stressing that an infringement of an athlete's personality rights amounting to a serious and clear violation of a fundamental right breaches public policy (8, § 207). The substantive content, in other words, was there (see also § 208). It was not applied, and commentary has made the same point in reproaching the Grand Chamber for not going further where it could have (68). A standard whose operation depends on whether a single supreme court will read its own public policy broadly protects less reliably than one binding every domestic organ *ex officio*. That is the difference the Inter-American model makes. The duty to ensure rights produces effects towards third parties in relations governed by private law, and a constitutional court of the region has already applied that horizontal effect to a football federation in order to hold it to due process.

None of this displaces the power of federations. Organisations keep a legitimate margin to set the conditions of competition, and no court is better placed than they are to fix rules of play or calendars. What changes is the burden of justification when a decision excludes someone from professional practice by invoking a conception of the value of sport. The technical appearance of the criterion then does not excuse the organisation from showing that the exclusion respects the dignity of the person affected and does not amount to discrimination. A regime built on open notions, whose application calls for evaluative choices and for decisions about who bears uncertainty, invites the scrutiny that any rule-making power attracts, and the private character of the deciding body does not reduce the weight of the rights its decision affects.

## 7 Institutional implications of the Convention standard

The effects of excluding an athlete or a club from professional competition go beyond their financial position. Exclusion also denies access to an activity that international instruments treat as a legally protected interest, and the dispute concerns more than private economic loss. The Grand Chamber made the same point about arbitration, noting that what is at stake in the sporting disputes the CAS examines may go beyond the pecuniary rights usually litigated in commercial arbitration and reach privacy, bodily and psychological integrity and human dignity (8, § 208). Review of federation decisions cannot, on that account, be left to the disposition of the parties or handled as the internal business of an association.

The International Charter of Physical Education, Physical Activity and Sport proclaims in Article 1 that the practice of physical education, physical activity and sport is a fundamental right for all, and Article 1.1 extends that right to every human being without discrimination on any ground, sexual orientation and social origin among them (17). The Inter-American order has taken an equivalent step in treaty form: the Inter-American Convention on Protecting the Human Rights of Older Persons recognises in Article 22 the right to recreation, physical activity, leisure and sport, and requires states parties to promote the corresponding services and programmes (69). That instrument covers one group only, so its value lies in showing that the region has begun to frame access to sport as an enforceable right rather than in grounding a general rule. Public health data support the same conclusion: physical inactivity is a leading risk factor for premature mortality and accounts for six per cent of deaths worldwide (18), which ties sporting practice to Sustainable Development Goal 3, on ensuring healthy lives and promoting well-being for all at all ages (70, 71).

Scholarship has not settled whether that interest amounts to an autonomous right. Jain (72) holds sport to be intrinsically constitutive of human dignity, which would call for recognition as a freestanding human right, while allowing that it may instead be derived from the settled rights to health, education, cultural life, work and non-discrimination, although the derived route requires proof that the parent right was violated. The argument made here does not turn on that question, for a reason drawn from the preceding section: dignity is already embedded in the applicable standards, in the Inter-American order and, on the Grand Chamber's own account, in the public policy of the arbitral seat. Access to professional sporting practice need only be a legally protected interest for its arbitrary denial to bring into play Articles 8 and 25 of the American Convention and the prohibition of discrimination.

Concrete duties follow for the legislature. A state cannot legislate its way out of obligations it has assumed: a party may not invoke the provisions of its internal law to justify failure to perform a treaty (73, art. 27), and Article 2 of the American Convention requires states to adopt the legislative measures needed to give effect to the rights recognised. Clauses conferring primacy on international federation rules over any domestic provision admit, on that basis, only an interpretation in conformity with the Convention, one that confines such primacy to the technical and organisational business of competition and leaves the guarantees of the Convention untouched; a statute read otherwise places the state in breach. Brazilian law shows what confinement of that kind looks like in positive terms. Prior recourse to sporting justice is required only for actions relating to discipline and competitions, and it lapses after sixty days; the general guarantee that no statute may exclude from judicial consideration any injury or threat to a right continues to apply (47, Articles 5, XXXV, and 217, §§ 1 and 2), and the tribunals of sporting justice must always secure the right to a full defence and the adversarial principle (48, Article 52). That arrangement produces a bounded priority rather than exclusivity: limited by subject matter, capped in time and conditional on the internal instance affording guarantees. Its limits are judicially enforced, as the Superior Labour Court showed when it held a pre-constitutional rule extending the exhaustion requirement to labour claims incompatible with the 1988 Constitution (50), and Brazilian scholarship reads sporting justice as a stage within the constitutional guarantee that no injury may be withdrawn from judicial consideration, rather than as an alternative to it (74).

Judicial organs receive an authorisation that regional practice has barely used. Conventionality control is exercised *ex officio* and at every level (33, § 225; 34, § 239), so an ordinary judge hearing a claim against a federation need not wait for the constitutional court before testing the applicable rule against the treaty. The same footing allows recognition or enforcement to be refused to sporting decisions taken without minimum guarantees, and the formal finality of an award is no obstacle, since *res judicata* does not cure the absence of fair proceedings. The duty reaches the enforcement stage as well. On the settled line running from *Muelle Flores* to *Teachers of Chañaral* (28, 29), effective enforcement mechanisms must be available against final decisions rendered against private parties as much as against state entities, and enforcement must be “complete, perfect, integral and without delay” (16, § 60).

Sporting organisations bear the consequence that matters most in practice. The burden of justifying an exclusion rests on whoever imposes it, so eligibility rules that remove someone from professional practice require a foundation that can be demonstrated in terms of dignity and non-discrimination. Internal procedures must meet the requirements of due process, and that includes a decision delivered while it can still serve the person affected. The Peruvian precedent shows how such an obligation fails through the design of the rules: a licensing regulation allowed enforcement to be suspended in favour of the penalised club and provided no equivalent remedy for the club harmed by that suspension. Non-interference, finally, loses its footing when raised against a state performing an international obligation, because the charge of political intrusion assumes a discretionary act of public power rather than the discharge of a duty under the Convention; state supervision of federation rules, as the Colombian Ministry of Sport exercises it, already coexists with organisational autonomy.

Taken together, these consequences present the model less as a restriction imposed on the sporting order from outside than as a condition of its legitimacy. An organisation that decides disputes with guarantees, gives reasons for its exclusions and accepts scrutiny strengthens the authority it claims; one that closes itself to all external review leaves its rules open to permanent challenge and hands the conflict to forums it did not choose. The sequence traced in this article, from Strasbourg and Luxembourg to the constitutional courts of the region, bears that out: resistance to review has cost the sporting order more than acceptance of minimum standards would have.

## 8 Discussion

The transnational sporting order decides disputes that determine the professional activity, the assets and the reputation of those subject to it, and it does so invoking an autonomy that has become a claim of immunity from judicial and constitutional review. This article asked whether that claim holds up against the obligations the American states assumed with regard to judicial guarantees, and what protection is owed to an athlete or a club when the organisation excluding them sits beyond the reach of the forum that might protect them.

Examination of the doctrinal construction showed that the order claiming closure describes itself as plural in its principles and hybrid in its method, since its decisions draw constantly on state law, regional law and human rights law. Autonomy, understood as a functional community's capacity to regulate itself, is not in dispute, and the reasons behind it deserve to be taken seriously: uniform rules, quick decisions and a short professional life are real concerns, and both European courts that might have rejected those reasons accepted them instead. Immunity from external review is a different proposition, and it follows from neither the fact of autonomy nor the reasons supporting it. The system itself belies that step when it counts procedural fairness among the principles it applies.

European case law established that such immunity can be limited and set a demanding standard of review where arbitral submission is imposed as a condition of professional practice. It went further than is usually noticed, describing sport governing bodies as exercising powers akin to regulation-making powers, holding that private entities not governed by public law regulate individual activity in fact, with the capacity to restrict rights, and concluding that imposition by a private body calls for more protection than imposition by law. The scheme of protection nonetheless remained tied to the territorial jurisdiction of the state where the award is challenged and to a restrictive notion of public policy, so the substantive allegations of discrimination went unexamined. EU law confirmed the same boundary by confining review of the award to conformity with public policy, and setting aside remains exceptional in practice.

The Inter-American order holds the elements that scheme lacks. Judicial guarantees attach to the materially adjudicative or punitive function, whatever the formal nature of the body exercising it. Horizontal effect reaches relations between private parties through the state's duty to ensure rights, and it has been worked out in contentious litigation over the enforcement of final judgments against private debtors rather than left to advisory reasoning. Conventionality control binds every public authority *ex officio*. Those elements combine into a standard whose operation rests on the obligations a state assumed towards those within its jurisdiction, irrespective of where the federation is domiciled.

Testing the model in four jurisdictions of the region confirmed both its viability and its present want of foundation. Brazil and Chile send contractual and employment disputes between player and club to the state courts, whether under a special labour regime or the ordinary labour jurisdiction, and Colombia reaches them through tutela, so that closure operates in disciplinary, eligibility and competition matters. Peru was the only jurisdiction examined that legislated the primacy of federation rules without confining it to those matters, and the collision surfaced in litigation. Its constitutional court, faced with the clause excluding recourse to the ordinary courts, held that nothing justifies relieving a federation of review where it disregards due process, and refused to accept spheres exempt under cover of private self-determination. The ruling reached the right result while dispensing with any grounding in the Convention, leaving the statutory clause of primacy intact and remaining open to being cast as state interference in a private organisation's affairs.

That comparison also disposed of the argument most often raised against external review. Speed matters in sport, and for the reason usually given, since careers are short and a remedy that arrives late is no remedy. In the two cases examined, the failure of speed came from the federation system itself: one federation body overran the time limits of its own statute, and another left final sanctions unenforced until the competition had moved on. One jurisdiction in the region has shown how the two concerns fit together, giving sporting justice priority in disciplinary and competition matters, capping that priority in time and leaving the general guarantee of access to a judge untouched.

The analysis of eligibility rules completed the demonstration. The notions grounding exclusions work as normative criteria of contested content and need a substantive conception of the value of sport before they can be applied, while decisions that look technical, such as who bears the burden of proof in disputes over prostheses, embody institutional choices with distributive consequences. Review confined to procedure does not satisfy the guarantee owed.

We maintain, then, that sporting sovereignty is a delegated and conditioned authority, and that in states parties to the American Convention the finality of the arbitral award and the doctrine of non-interference yield to the minimum guarantees of Articles 8 and 25 through conventionality control. The Inter-American standard protects more broadly than the European one in its material scope, in its rule of attribution and in its operability, for the reasons developed in the fourth section.

The contribution proposed is an interpretive criterion of immediate application. Sporting autonomy is legitimate where it pursues a permissible aim, observes procedural guarantees in every decision bearing on rights, delivering it while it can still serve the person affected, and respects the substantive limit of dignity and non-discrimination. Statutory clauses conferring primacy on international federation rules admit only an interpretation in conformity with the Convention, one that confines such primacy to discipline and the organisation of competitions, subjects it to a defined time limit and leaves everything else to the courts. The ordinary judge is empowered to review *ex officio* and to refuse enforcement to decisions taken without minimum guarantees. And the burden of justifying an exclusion falls on the organisation imposing it, the technical appearance of the criterion being no substitute for showing that the exclusion is compatible with the dignity of the person affected.

The region holds a standard more protective than the one applied so far in sports arbitration, and its courts have not used it. Athletes have begun to invoke the American Convention before domestic courts, yet the judgments that subjected federations to constitutional review reached the right outcome through domestic law alone, which leaves their reasoning more exposed than it need be before international bodies inclined to treat any state act as interference. Invoking the Convention standard moves the basis of the decision from the will of one state to an obligation shared by all the states of the region, and removes the dispute from the terrain on which the threat of competitive exclusion keeps its force. Rebuilding the legitimacy of the sporting order begins with recognising that its authority is exercised over persons who hold rights and who do not lose that status by entering a competition.

## Data Availability

The original contributions presented in the study are included in the article/Supplementary Material, further inquiries can be directed to the corresponding authors.
